# Temporal Dynamics of the Soil Metabolome and Microbiome During Simulated Anaerobic Soil Disinfestation

**DOI:** 10.3389/fmicb.2019.02365

**Published:** 2019-10-15

**Authors:** Shashika S. Hewavitharana, Emmi Klarer, Andrew J. Reed, Rachel Leisso, Brenton Poirier, Loren Honaas, David R. Rudell, Mark Mazzola

**Affiliations:** ^1^Department of Plant Pathology, Washington State University, Wenatchee, WA, United States; ^2^United States Department of Agriculture-Agricultural Research Service, Physiology and Pathology of Tree Fruits Research, Wenatchee, WA, United States

**Keywords:** community metabolism, microbial network, metabolic network, hydrocarbon pathway, syntrophy, soil microbiome, soil metabolome, anaerobic soil disinfestation

## Abstract

Significant interest exists in engineering the soil microbiome to attain suppression of soil-borne plant diseases. Anaerobic soil disinfestation (ASD) has potential as a biologically regulated disease control method; however, the role of specific metabolites and microbial community dynamics contributing to ASD mediated disease control is mostly uncharacterized. Understanding the trajectory of co-evolutionary processes leading to syntrophic generation of functional metabolites during ASD is a necessary prelude to the predictive utilization of this disease management approach. Consequently, metabolic and microbial community profiling were used to generate highly dimensional datasets and network analysis to identify sequential transformations through aerobic, facultatively anaerobic, and anaerobic soil phases of the ASD process and distinct groups of metabolites and microorganisms linked with those stages. Transient alterations in abundance of specific microbial groups, not consistently accounted for in previous studies of the ASD process, were documented in this time-course study. Such events initially were associated with increases and subsequent diminution in highly labile metabolites conferred by the carbon input. Proliferation and dynamic compositional changes in the Firmicutes community continued throughout the anaerobic phase and was linked to temporal changes in metabolite abundance including accumulation of small chain organic acids, methyl sulfide compounds, hydrocarbons, and *p*-cresol with antimicrobial properties. Novel potential modes of disease control during ASD were identified and the importance of the amendment and “community metabolism” for temporally supplying specific classes of labile compounds were revealed.

## Introduction

Anaerobic soil disinfestation (ASD) is a practice that has been successfully employed for the control of several plant pathogens in a variety of cropping systems (Rosskopf et al., 2015). Field application of ASD involves a sequence of steps comprised of the incorporation of labile organic matter, irrigation of soil to field capacity, and covering the treated soil with an oxygen impermeable film (Blok et al., 2000; Shinmura, 2000). Utilization of ASD is relevant to organic agriculture and its application has increased in conventional production systems under conditions where there are no effective chemical soil fumigants or where their use is subjected to regulatory restrictions.

Significant effort has been targeted toward enhancing ASD efficacy for soil-borne disease control with an emphasis on optimizing implementation variables such as carbon source (Butler et al., 2012; Hewavitharana et al., 2014; Serrano-Pérez et al., 2017; Shrestha et al., 2018), irrigation volume and duration (Daugovish et al., 2015), length of the incubation period, and the effects of soil temperature (Hewavitharana et al., 2015). Concurrent studies have examined the influence of these variables on the proposed mechanisms that yield disease suppression in response to ASD. Prior investigations focused primarily on the role of soil volatile fatty acids (VFA; Momma et al., 2006) and functional changes in soil microbial community structure during the ASD process (Liu et al., 2016). Additional volatile compounds including dimethyl trisulfide, 2-ethyl-1-hexanol, decanal, and nonanal are produced in a carbon resource-dependent manner during ASD and were demonstrated to possess biocidal activity (Hewavitharana et al., 2014).

Disease suppression also has been associated with distinct changes in the soil microbiome that are elicited in an ASD carbon resource-dependent manner (Hewavitharana and Mazzola, 2016; Liu et al., 2016; Poret-Peterson et al., 2019). Multiple studies have monitored changes in soil bacterial community structure that occur in response to ASD treatment. However, monitoring of microbiome structural shifts has been limited to broad changes in bacterial community in pre- and post-treatment soil and did not examine temporal dynamics of both bacteria and fungi at a more refined level (Mowlick et al., 2013b; Stremińska et al., 2014; Liu et al., 2016). An initial increase in abundance of Firmicutes has been repeatedly observed in ASD treated soils (Momma et al., 2010; Mowlick et al., 2012; Stremińska et al., 2014) and the role of these bacteria in disease control has been implied due to their capacity to produce volatile fatty acids (Rosskopf et al., 2015).

Although studies have addressed production of certain metabolites in soils resulting from anaerobic respiration, such as VFA (Momma et al., 2006), imposing a metabolomics approach to investigate the sequential generation of metabolites during the ASD process may clarify the suite of compounds that are potentially involved in pathogen suppression or that may modulate shifts in the soil microbiome that ultimately result in disease control. Jones et al. (2014) coined the term “community metabolism” to specifically recognize studies in which the naturally occurring products of metabolism from the entire community of a given sample are analyzed simultaneously. Metabolic biomarkers can be linked to a given environmental exposure such as ASD to predict the status of the ecosystem; potential for disease suppression in this case. The advantage of biomarker profiling compared to the assessment of a single biomarker is that the former provides opportunity to identify relevant biological response(s) as a result of a particular exposure, while the latter affords only an estimate of the potential response of the ecosystem to a given stimuli. Untargeted metabolomics can be employed in biomarker profiling and is of value particularly in a system that has not been extensively surveyed such as ASD treated soil. Since untargeted metabolomics does not rely upon a spectrum of *a priori* selected metabolites, it is robust in discovering novel metabolites (Baig et al., 2016). Follow-up targeted metabolomics studies can be used to explore deeply into any aspect that seems to be important based on the untargeted analysis. Such methods have previously been used in assessing factors that influence postharvest fruit quality (Rudell et al., 2008; Leisso et al., 2013, 2015), soil pollution (Hernandez-Soriano and Jimenez-Lopez, 2014; Jones et al., 2014) and identifying targets that predict progression of human disease and treatment response (Boca et al., 2016).

One of the approaches that stem from metabolic or biomarker profiling is building metabolic correlation networks to link biological functions and biochemical pathways (Steuer et al., 2003). By employing a metabolomics approach, the complexity of metabolic networks is grasped via comprehensive characterization of small-molecule metabolites in biological systems and the manner in which they change in response to a variety of stimuli (Jones et al., 2013). Metabolic correlation networks can be generated based on the pair-wise correlation between respective concentrations of metabolites in a given sample (Weckwerth and Fiehn, 2002). Furthermore, metabolites can be grouped into metabolic modules by combining correlation analysis with distance metric and clustering methods such as weighted correlation network analysis (WGCNA; Langfelder and Horvath, 2008).

Anaerobic phase of ASD induces a distinct shift in the soil microbial profile in a carbon source dependent manner (Hewavitharana and Mazzola, 2016) which results in a distinct metabolic profile. Some of these metabolites may be utilized as biomarkers of dominant microbial groups that are recruited in response to ASD. Other metabolites may have functions in energy and carbon transfer through the trophic levels in soil and successions of microbial consortia which may be linked to soil physiochemical changes such as soil reduction. Certain soil metabolites derived in the ASD process act to directly inhibit plant pathogens (Hewavitharana et al., 2014; van Agtmaal et al., 2015) and volatile molecules produced during ASD may function to induce systemic resistance in host plants (Naznin et al., 2014).

To our knowledge, comprehensive analysis of the soil metabolome, including classification of the polar, non-polar and volatile metabolites, generated during the course of ASD and concurrent assessment of changes in the soil microbiome, has not been reported. Our hypothesis was that these sequential transformational events lead to temporal production of certain soil metabolites in ASD treatment (with rice bran as the carbon source) involved in induced plant systemic resistance or may act to directly inhibit plant pathogens resulting in soil-borne disease control. The objectives of this study were to (i) document changes in the soil community metabolome as a response to the perturbation of ASD, (ii) identify marker metabolites that indicate presence of successional microbial groups that may provide the disease suppression trait in soil, (iii) classify metabolic modules that may have functional significance, and (iv) identify metabolomic networks that may reveal novel pathways of anaerobic soil metabolism.

## Materials and Methods

### Plant Bioassay for Demonstration of ASD Efficacy in Fusarium Wilt Control

Soil was obtained from the root zone of apple trees established at the Washington State University Columbia View (CV) Research orchard (Orondo, WA, United States; latitude 47.56235 N, longitude 120.24499 W) in autumn of 2014. The dominant soil type of this site is silt loam (30% sand, 9% clay, 61% silt). An initial experiment was conducted to assess the effect of ASD with and without a carbon input on control of the target pathogen, *Fusarium oxysporum* f. sp. *fragariae*, causal agent of Fusarium wilt of strawberry. The experiment consisted of four treatments; a no treatment control, rice bran only soil amendment, irrigation of soil to field capacity with no carbon amendment to the soil (ASD-NA) and ASD conducted with rice bran (RB) as the carbon input. Soil was mixed in a cement mixer for 10 min and 2.20 kg samples were placed in 3 L plastic pots. Soils were inoculated with a *F. oxysporum* f. sp. *fragariae* conidial suspension to attain a concentration of 300 cfu/g soil and incubated for 24 days in an environmental growth chamber set to 24°C (12 h with supplemental lighting) and 18°C (12 h without supplemental lighting). For the ASD with RB treatment, RB (34.5 g per pot equivalent to 20 Mg per ha per 15 cm depth in the field) was homogeneously mixed into soil. Estimated nutrient concentration of RB was *C* = 47.8, *N* = 2.49, *P* = 1.53, *K* = 1.60, and *S* = 0.18%, C:N ratio = 19:1, pH = 6.2 (Soiltest Farm Consultants, Inc., Moses Lake, WA, United States). Soils treated with ASD received 792.0 mL of distilled water to saturate the soil to field capacity (−12.5 kPa) and were sealed in gas impermeable Bitran^®^ bags (Com–Pac International, Carbondale, IL, United States). ASD treatment lasted 15 days, during which aerobic soils received 100 mL of distilled water two times per week to maintain moisture levels. Pots were removed from bags at the end of the ASD treatment period and one strawberry crown of the cultivar Albion was planted in each pot 35 days post-treatment, with six replicate pots per soil treatment. Plants were grown under a 12 h photoperiod with a 28°C/20°C day/night temperature regime and harvested after 82 days at which time soil and plant crown samples (six per treatment) were collected and were stored at −80°C until used in extraction of DNA.

DNA was extracted from 0.25 g of soil using a DNeasy PowerSoil DNA isolation kit (Qiagen, Germantown, MD, United States) and from 50 mg of ground crown tissue sample using a DNeasy PowerPlant Pro DNA isolation kit (Qiagen). The quantity of the inoculated pathogen was assessed by real-time quantitative PCR using *F. oxysporum* f. sp. *fragariae* specific primers (FofraF and FofraR) and reaction conditions as previously described (Suga et al., 2013) on a Step One Plus Real-Time PCR system (Applied Biosystems, Waltham, MA, United States).

### Soil Preparation, Treatments, and Incubation Conditions for Metabolome and Microbiome Analysis

**FIGURE 1:**
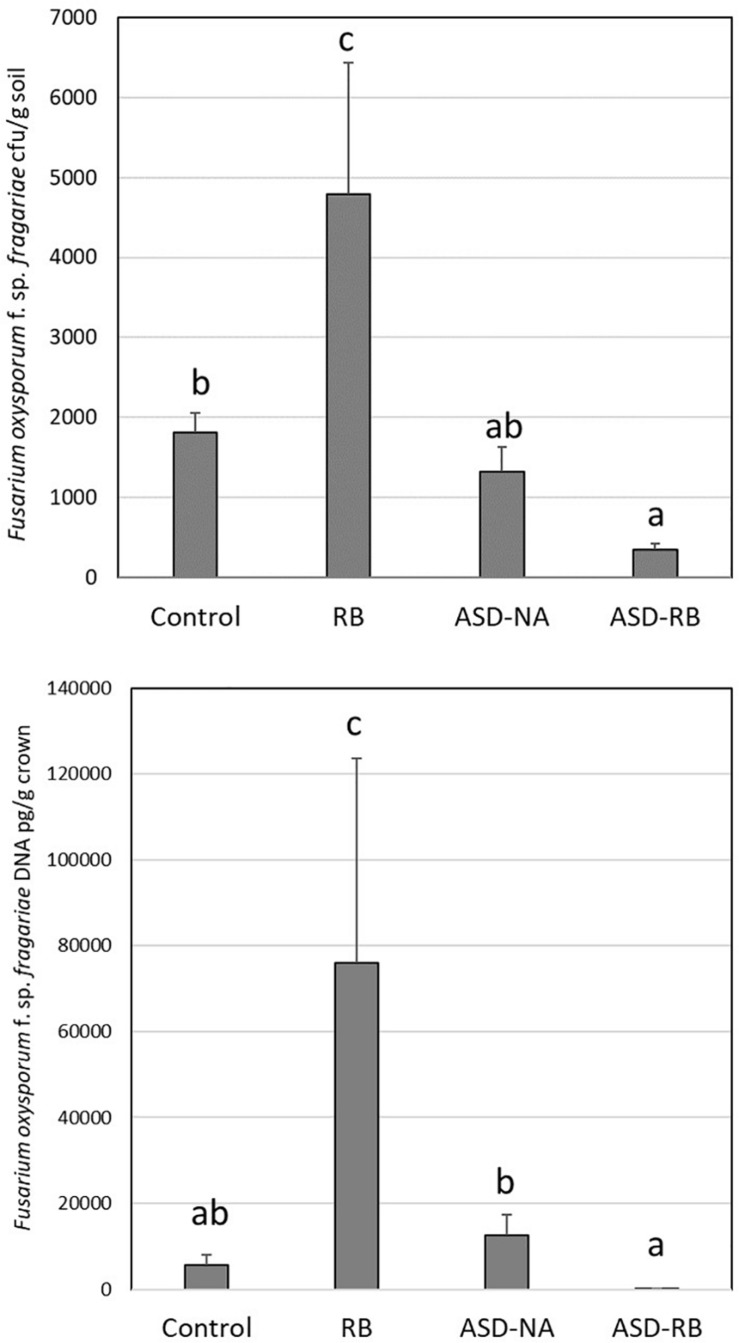
Effect of anaerobic soil disinfestation (ASD) with rice bran amendment (20 t ha^–1^; ASD-RB), ASD conducted without a carbon amendment (ASD-NA), and rice bran soil amendment (20 t ha^–1)^ conduct under aerobic conditions (RB) on *Fusarium oxysporum* f. sp. *fragariae* soil density (top panel) and strawberry crown infection by this wilt pathogen (bottom panel). Values represent the mean colony forming units of the pathogen g^–1^ soil and quantity of pathogen DNA (μg g^–1^ crown tissue) with *n* = 6, respectively. Data were analyzed using one-way ANOVA. Means designated with the same letter are not significantly different (*P* > 0.05) as determined by Tukey’s honest significant test. Error bars represent standard error of the mean with *n* = 6.

Columbia View orchard soil was passed through a 4-mm sieve and 100 g samples were placed into 487 mL microcosms (glass jars). Jar lids were perforated to make a 1 cm diameter hole into which a rubber septum was inserted. The experiment consisted of two treatments; ASD amended with rice bran (RB) and a no-carbon input ASD control (ASD-NA), seven time points; day 0, 1, 2, 3, 7, 11, and 15, with four samples per treatment at each incubation duration point. In total, across all treatments and time points, 56 samples analyzed. For the purposes of this study, RB soil amendment treatment was not included as pathogen suppression (e.g., *F. oxysporum* f. sp. *fragariae*) was not realized under such conditions (Figure 1). In addition, RB soil incorporation does not yield an anaerobic environment (Mazzola et al., 2018), nor result in amplification of target microbial populations commonly associated with ASD-induced disease suppression, such as *Clostridium* spp. For the ASD treatment, 1.57 g of RB (equivalent to 20 Mg per ha per 15 cm depth in the field) was homogeneously mixed into soil. Estimated nutrient concentration of RB was *C* = 47.8%, *N* = 2.49%, *P* = 1.53%, *K* = 1.60%, *S* = 0.18%, C:N ratio = 19:1, pH = 6.2 (Soiltest Farm Consultants, Inc., Moses Lake, WA, United States). For both treatments, 25 mL of distilled water was added to saturate the soil pore spaces. The headspace volume over the microcosms was 409 mL. Jars were incubated in an environmental growth chamber for 15 days at 24/18°C with 12-h photoperiod. Photosynthetically active radiation (PAR, 400–700 nm) just above the jars was 226 μmol m^–2^ sec^–2^. Immediately after initiating the experiment, headspace volatiles were extracted, and a representative soil sample was collected using a #3 cork borer (7 cores from each microcosm). Soil samples were placed in a chilled sample cup, followed by immediate immersion in liquid nitrogen and stored at −80°C. This procedure was followed for soil sampling at all incubation duration points. Frozen soil cores were cryogenically milled to a fine powder and stored again at −80°C before use for metabolite analysis. For day 0 and day 1 time points, headspace O_2_ and CO_2_ composition was analyzed every 4 h and once for the remaining incubation duration points as described below.

### Metabolite Extraction and Evaluation

Frozen soil samples were analyzed using three extraction protocols combined with 3 instrumental analyses to evaluate the soil metabolome. Extractions were conducted replicate by replicate to minimize extraction artifacts. Headspace O_2_ and CO_2_ percentage in the microcosms was analyzed using a handheld gas analyzer (Dansensor A/S, Ringsted, Denmark).

#### Volatile Metabolite Analysis

50 mL and 500 mL of headspace, metered using a soap film bubble flowmeter, was vacuumed through using a pump (Universal electric motor 115 V, 60 Hz, Magnetek^®^, Menomonee, WI, United States) and adsorbed onto two separate 60 mm (length) × 6 mm (o.d.) silanized glass traps packed with Tenax^®^ TA porous polymer (60/80 mesh) connected in series following the trap. Air was returned to the sample. Untrapped volatiles were removed from the return air stream by filtering using another trap filled with a combination of Tenax^®^ and Carbosieve 3 (60/80 mesh) (Supelco, St. Louis, MO, United States).

Analyte was desorbed, cryofocused and analyzed using an Agilent (Santa Clara, CA, United States) 6890/5975 gas chromatograph/mass spectrometer equipped with a Gerstel (Baltimore, MD, United States) Multipurpose Sampler (MPS), Dynamic Headspace Sampler (DHS), and Thermal Desorption Unit (TDU). Water was removed using the DHS to sweep traps with 400 mL of He at 40 mL min^–1^ while the trap was maintained at 35°C. Traps were desorbed, cryofocused, introduced into the GC and analyzed as outlined by Rudell et al. (2017).

#### Trimethylsilyl (Oxime) Derivative Analysis of Polar Metabolites

Analyte in frozen powdered soil samples (2.0 g) were extracted using 50% methanol in water as described by Swenson et al. (2015) followed by trimethylsilyl (oxime) [TMSO] derivatization and GC-MS analysis conducted as essentially described in Rudell et al. (2008).

#### Non-polar Metabolite Analysis

Two grams of frozen soil powder was weighed into a 15-ml polypropylene centrifuge tube previously chilled in liquid N_2_. Tubes were transferred onto ice and 100 μl of α-tocopherol acetate internal standard followed by 5 ml of 2:1 acetone: 0.2 M HEPES pH 7.7 buffer were added. Capped tubes were vigorously shaken by hand for 1 min and sonicated for 10 min in a 30°C water bath. Samples were centrifuged at 2000 rpm for 5 min and the supernatant (first fraction) was poured into 16 × 125 mm borosilicate test tubes. Four milliliters acetone was added to the pellet and vigorously shaken for 1 min to break the pellet and ultrasonicated for 10 min followed by centrifugation at 2000 rpm for 4 min. Supernatant was carefully combined with the first fraction. This step was repeated once with acetone and then with 2 mL hexanes. Acetone and hexanes supernatant were combined with existing supernatant and transferred to a new polypropylene centrifuge tube. The tube was vortexed for 1 min, centrifuged at 2000 rpm for 2 min, and the hexanes fraction transferred into a clean borosilicate tube (13 × 100 mm). Partitioning with hexanes was repeated twice using 2 ml then 1 ml. Hexanes phases were combined and dried under a gentle stream of N_2_ (g) for approximately 30 min, the residue dissolved in 250 μl acetone by sonicating for 10 min, and finally filtered using a 0.45 μm PTFE (Whatman, Maidstone, United Kingdom) syringe filter prior to analysis.

#### Liquid Chromatograph-Mass Spectrometry Quadrupole Time-of-Flight

The analysis was performed as described by Leisso et al. (2015) monitoring positive ions.

#### Quality Control

Three reference samples of soil were analyzed along with the test samples daily using GC-MS (trimethylsilyl (oxime) derivative analysis) and LC-MS sequences and assessments were made as previously described (Rudell et al., 2009). The reference sample consisted of a bulk sample of “ASD-day 5” soil that had been incubated, sampled, frozen, and ground as described for test samples.

### Data Acquisition, Deconvolution, and Peak Identification

For volatile and polar metabolite data, user-defined GC-MS and LC-MS libraries were prepared as described by Rudell et al. (2008, 2009) and non-polar as described by Rudell et al. (2017) to search for unique components within the chromatographic data. Peak retention indices (RI) were generated using the same method. List of compounds, RI and target ions are presented in Supplementary Table S1. Mass spectral tags (MSTs) cataloging and compound identification were also performed as described by Rudell et al. (2009).

Non-polar metabolite data analyzed by LC-MS QTOF were acquired by MassHunter Data Acquisition software and converted to the ^∗^mzdata format using MassHunter Qualitative Analysis software. Data were imported into MZmine 2 preprocessing software (Pluskal et al., 2010). The minimal mass spectral noise (1000) was removed from each sample, and ion chromatograms were generated from a mass window of 0.1 m/z and minimum time span of 0.1 min and minimum height of 1000. The chromatograms were smoothed using Savitsky-Golay smoothing with a filter width of 7 scans. The smoothed chromatograms were deconvoluted using local minimum search with a 0% chromatographic threshold, a 0.05 min retention time range between adjacent peaks, a 1000 minimum peak height, ratio of 1:1 peak apex to peak edge intensity, and a peak duration range of 0.1 to 3 min. Peaks of the deconvoluted smoothed chromatograms were identified with a custom database based on m/z and retention time (RT). The peaks of every sample were aligned and combined into a single peak list. Peaks were checked individually to assure consistent peak choice and accurate identification assignment.

### Statistical Modeling and Analyses of Metabolite Data

Raw peak area data were corrected by comparison to that of phenyl β-D-glucopyranoside, α-tocopherol acetate for the TMSO protocol and non-polar metabolite analysis protocol, respectively, in each sample and then by soil weight.

The metabolome was initially assessed using principal components analysis (PCA) performed on mean centered data divided by standard deviation [MetaboAnalyst 3.0 (Xia and Wishart, 2016)]. Heatmaps of metabolomics data were generated using Euclidean distance measure and Ward clustering algorithm. Replications remained separate for modeling using the Non-linear Iterative Partial Least Squares algorithm (NIPALS) employed by Unscrambler X (Camo, Trondheim, Norway).

### Statistical Modeling and Analyses of Metabolic Data

“Undirected network” was used for the network generation as the metabolites are derived from the multi-genomic soil community residing in a heterogeneous matrix that is highly complex and has the probability of reverse and abiotic reactions occurring rather than using “directed network” which is a feature of metabolic networks of single organisms where many enzyme-catalyzed reactions are irreversible (Barabási and Oltvai, 2004). In the current study, based on power analysis, the metabolic network satisfied scale-free criterion.

Node clustering as well as node and module proximity are indicative of inter-relationships among nodes and modules within the larger network. Modules of highly correlated metabolism within the ASD treatment were identified using the R package weighted correlation network analysis (WGCNA; Langfelder and Horvath, 2008). Polar, non-polar, and volatile metabolite compounds were analyzed collectively. Signed, weighted metabolite co-abundance correlation (Pearson correlation) networks were calculated using all examined incubation duration points and replicates. A scale free topology was assumed for the network using soft threshold β = 10 based on a power analysis. Clusters were identified using automatic module detection via dynamic tree cutting algorithm, using minimum module size 30. Cluster “eigengene” or metabolite, in this case, is defined as the first principal component of the metabolite abundances across replicates of each sample point (Langfelder and Horvath, 2008). Similar clusters were merged if the Pearson correlation between each cluster’s eigengene exceeded tree cut height of 0.15.

### Microbial Community Analysis

Microbial populations were analyzed by amplicon sequencing of fungal and bacterial communities of DNA extracted from 0.25 g of frozen soil samples stored at −80°C using the PowerSoil DNA isolation kit (MO BIO Inc., Carlsbad, CA, United States). Amplicon sequencing and data processing were performed as described in Hewavitharana and Mazzola (2016). Specifically, the primer pair 515f (5′ -GTG CCA GCM GCC GCG GTA A-3′) and 806r (5′- GGA CTA CHV GGG TWT CTA AT- 3′) (Caporaso et al., 2011) were used to amplify the V4 region of the bacterial 16S ribosomal RNA gene. ITS1F-Bt1 (5′-TCC GTA GGT GAA CCT GCG G-3′) and ITS4Rbt (5′-TCC TCC GCT TAT TGA TAT GC-3′) primers were utilized for amplification of the fungal ITS region (White et al., 1990). The forward primer, of the above indicated primer pairs, was used to add sample specific barcodes. The PCR reaction conditions applied were 94°C for 3 min; 28 cycles of 94°C for 30 s, 53°C for 40 s, and 72°C for 1 min; with a final elongation at 72°C for 5 min and visualized on a 2% agarose gel for verification of the amplicon size. Based on molecular weight and DNA concentration multiple samples were pooled together in equal proportion. These were purified using Ampure XP beads (Agencourt Bioscience Corporation, Beverly, MA, United States). Pooled and purified PCR products (10 ng from each sample) were next used for the preparation of a DNA library according to the Illumina TruSeq DNA preparation protocol (Illumina, San Diego, CA, United States). MR DNA laboratory (Shallowater, TX, United States) performed all sequencing using Illumina MiSeq platform. Sequence data were processed using MR DNA analysis pipeline. Sequences were joined, depleted of barcodes followed by removing sequences shorter than 150 bp and sequences with ambiguous base calls. Sequences were denoised, Operational taxonomic Units (OTUs) generated and chimeras removed. Operational taxonomic units were defined by clustering at 3% divergence (97% similarity; Dowd et al., 2008, 2011; Edgar, 2010). Final OTUs were taxonomically classified using BLASTn against a curated database derived from RDPII^1^ and NCBI^2^. Sequences were deposited in the GenBank SRA database under Bioproject number PRJNA561262. Relative similarity in bacterial and fungal communities among treatments was assessed by performing ANOSIM and ordination of OTU data. Ordination plots based upon non-metric multidimensional scaling of OTU data were generated using PAST statistical package (Hammer et al., 2001).

### Statistical Modeling and Analyses of Microbial Data

As WGCNA analysis does not allow for excessive zero values in the current time-series data set, OTU data were subjected to filtration as follows. Number of replicates of each incubation duration point possessing OTU counts greater than 2 were determined for each OTU. Number of time points that comply with the above argument was determined for each OTU and filtered to remove OTUs that did not have at least one incubation duration point with counts greater than 2 across four replicates. Zero counts were replaced by 0.5, which corresponded to one-half of the smallest value in the data set. WGCNA analysis and network visualization was carried out as described above with soft threshold β = 14 based on power analysis, and tree cut height of 0.45. List of OTUs, count data, taxonomical classification, and microbial module designation are presented in Supplementary Table S2.

Metabolic and microbial networks were uploaded to Cytoscape software (Version 3.4; Shannon et al., 2003) with module assignments (colors) as the node attribute. Allegro Edge-Repulsive Fruchterman-Reingold layout (AllegroViva Corp., Santa Clara, CA, United States) was used for network spatial arrangement according to the edge weights calculated using the WGCNA.

## Results

### Metabolic and Microbial Profiles Defined Phases During ASD Treatment

**FIGURE 2:**
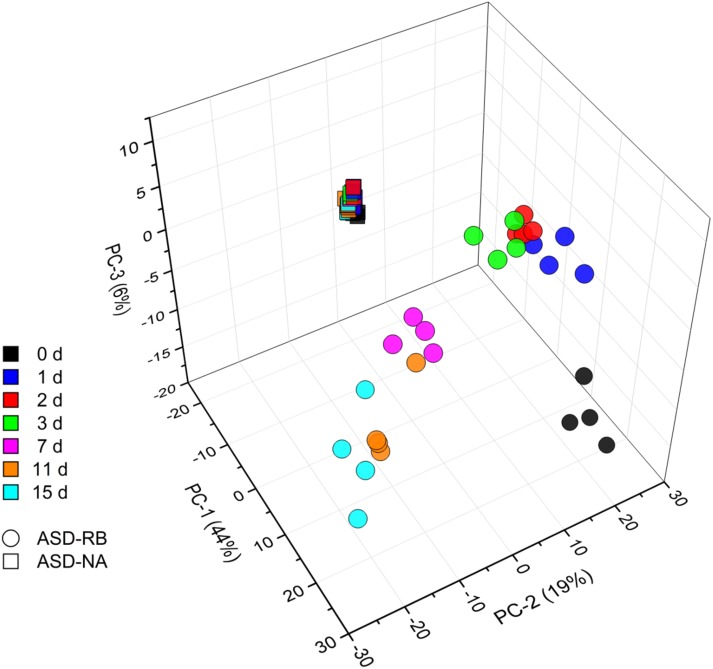
Principle component analysis scores plot representing changes of the soil community metabolome during a 15-d simulated anaerobic soil disinfestation (ASD) treatment using rice bran (20 t ha^–1^) as the carbon source (ASD-RB) and ASD conducted without the carbon amendment (ASD-NA). Levels of 691 metabolites are summarized for each observation. Metabolomic changes over incubation duration of the ASD with rice bran treated soil accounted for the vast majority of variance where the non-amended ASD metabolome changed little.

**FIGURE 3:**
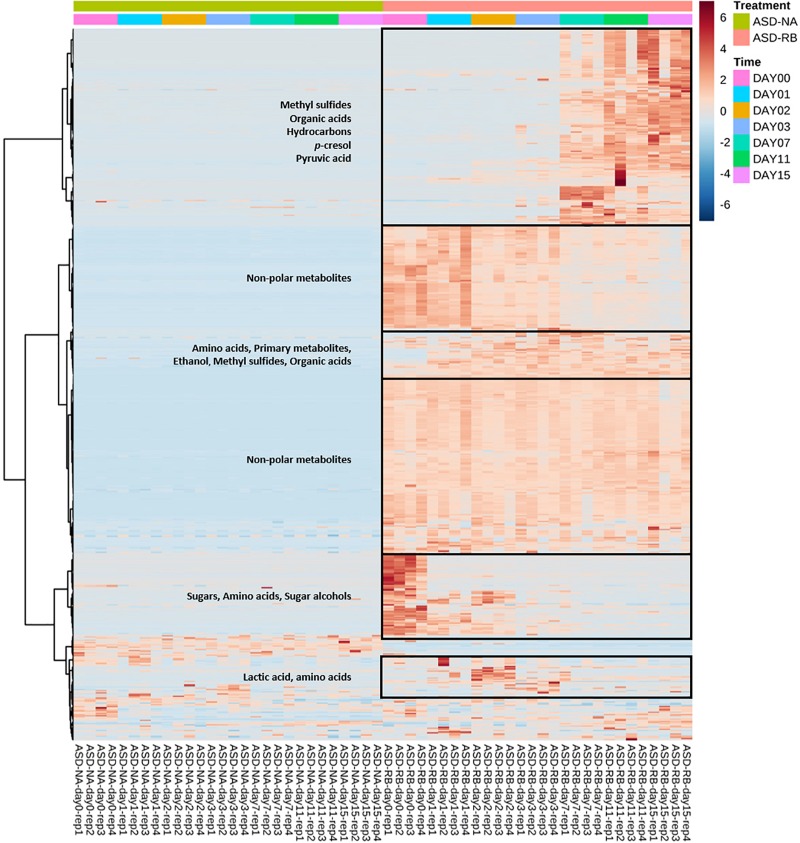
Heat map illustrating relative abundance of significant soil metabolites as influenced by soil treatment and time. Important groups of soil metabolites were identified in the boxes. Soil metabolites were clustered based on two-way ANOVA (adjusted *p*-value cutoff 0.05 and false discovery rate as multiple testing correction method) with repeated measures within subjects using the soil treatment and time series factors and their interaction to generate a heat map visualization with Euclidean distance measure and Ward clustering algorithm. Heatmap was generated using MetaboAnalyst software. ASD-NA, anaerobic soil disinfestation (ASD) conducted without a carbon source; ASD-RB, ASD using rice bran as the carbon source at a rate equivalent to 20 t ha^–1^ for 15 cm depth.

**FIGURE 4:**
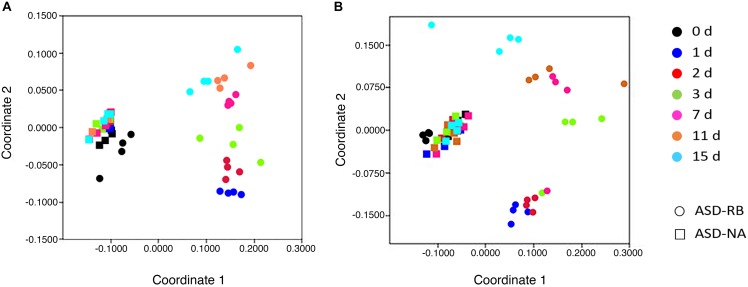
Relative similarity of **(A)**, bacterial and **(B)**, fungal communities during a 15-d simulated anaerobic soil disinfestation (ASD) treatment using rice bran (RB; 20 t ha^–1^) as the carbon source compared to ASD conducted without the amendment (ASD-NA). Microbial communities, when evaluated at the operational taxonomic unit level, showed highly dynamic changes over time in the ASD-RB treated soil, which were not evident when examined at the phylum level and was essential in detecting significant and protracted changes in community structure over the ASD incubation period. Ordination of soil microbiomes was conducted by non-parametric multidimensional scaling of operational taxonomic unit data using the Bray-Curtis similarity coefficient.

Sequential changes in the metabolome and soil microbial community defined the aerobic (phase 1), facultatively anaerobic (phase 2), and anaerobic (phase 3), respectively. The consumption of labile carbon drove O_2_ consumption leading to the anaerobic conditions in the ASD-RB treatment distinguishing it from ASD conducted without the carbon amendment. The addition of a carbon source contributed the majority of the variance to a PCA model comprised of the ASD-RB and ASD-NA metabolomes (44.2%) (Figure 2). Accordingly, metabolic (Figure 3), bacterial community (Figure 4A) and fungal community (Figure 4B) profiles were dynamic in ASD-RB treated soil but did not vary over time in ASD-NA soil based on ANOSIM.

### Changes in Headspace O_2_ and CO_2_

**FIGURE 5:**
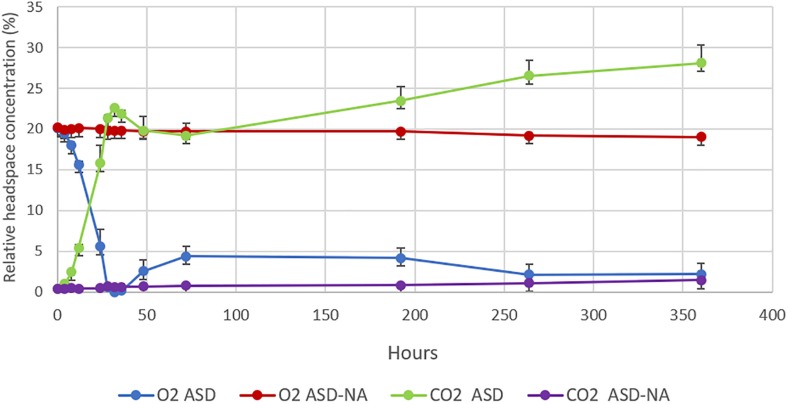
Changes in O_2_ and CO_2_ concentrations in the headspace of microcosms over the period of 350 h (15 days). O_2_ and CO_2_ concentrations were monitored in anaerobic soil disinfestation (ASD) treated soils with (ASD-RB) and without (ASD-NA) rice bran soil amendment. ASD was conducted using rice bran as the carbon source at a rate equivalent to 20 t ha^–1^. Values represent mean O_2_ and CO_2_ percentages in the headspace of microcosms of each treatment. Error bars represent standard error of the mean with *n* = 4.

Dynamics of relative concentration of O_2_ and CO_2_ in the headspace of jars for ASD-RB and ASD-NA treatments were markedly distinct (Figure 5). Average O_2_ concentration decreased from 20.1 to 0% within the first 32 h of incubation with a corresponding increase in CO_2_ from 0.43 to 22.58% in the ASD-RB treatment. In contrast, the headspace for the ASD-NA treatment exhibited minor deviations in both O_2_ (20.25 to 19.8%) and CO_2_ (0.33 to 0.6%) concentration during the same incubation duration period. After 72 h, O_2_ levels were maintained below 4.4% and CO_2_ levels were above 19.2% for the ASD-RB treated soil system for the duration of the incubation period.

### Metabolite and Microbial Correlation Networks

**FIGURE 6:**
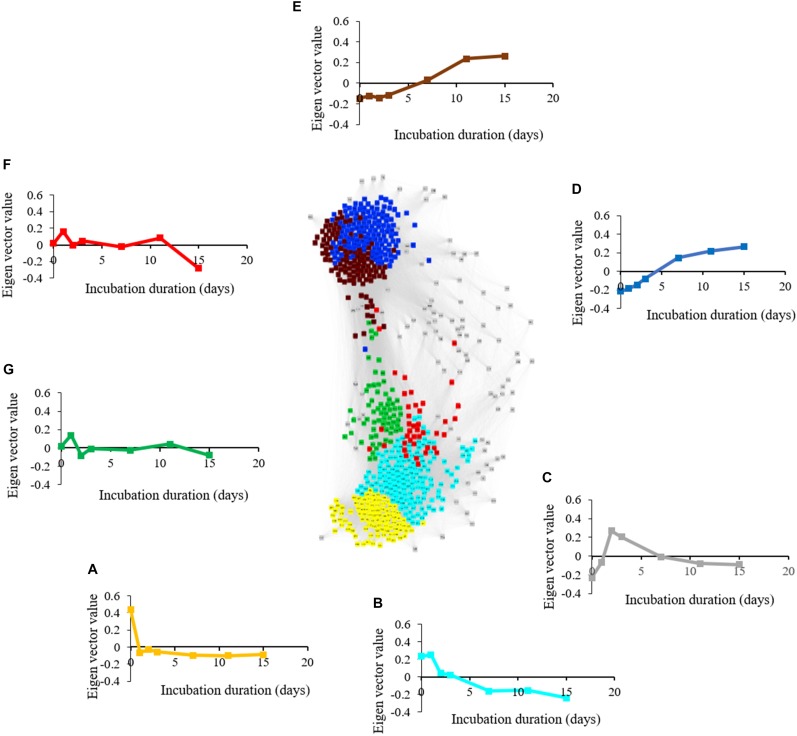
Weighted correlation network (WGCNA) and change in Eigen vector value of individual consumption, production, and intermediate metabolic modules revealing metabolic transition during simulated anaerobic soil disinfestation (ASD). Metabolite consumption modules included **(A)** (yellow: primarily amino acids and sugars) and **(B)** (turquoise: primarily non-polar metabolites including triacylglycerides). Metabolite intermediate modules were comprised of **(C)** (gray: unassociated metabolites; pyruvic acid, lactic acid, amino acids, dimethyl sulfide), **(F,G)** (fluctuating trends with unknown functional significance). Metabolite production modules included **(D)** (blue: primarily organic acids and alcohols) and **(E)** (brown: primarily hydrocarbons). ASD was conducted using rice bran as the carbon source at a rate of 20 t ha^–1^. Weighted gene correlation network analysis (WGCNA) was used to generate signed network using soft threshold power = 10. Cytoscape Allegro Edge-Repulsive Fruchterman-Reingold layout was used to visualize the network. Nodes representing metabolites with highly correlated levels are the same color.

**FIGURE 7:**
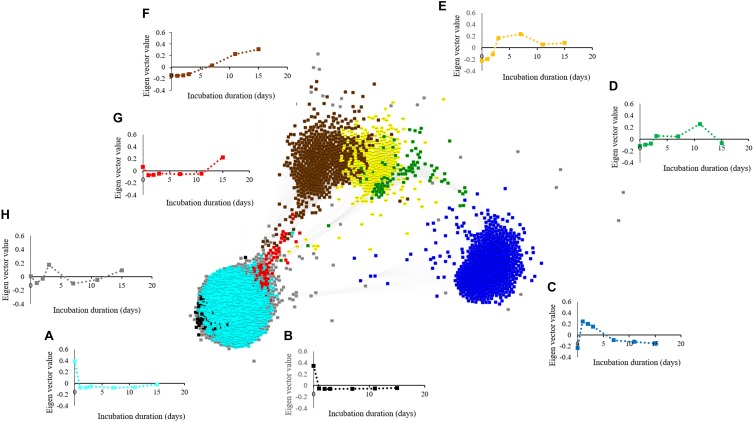
Weighted correlation network (WGCNA) and change in Eigen vector value of individual microbial decline, proliferation, and intermediate modules revealing changes in the microbiome during simulated anaerobic soil disinfestation (ASD). Microbial decline modules included **(A)** (turquoise:dominant with aerobic bacterial [*Bacillus* spp., *Pasteuria* spp.] and fungal phyla [*Penicillium* spp., *Acremonium* spp.]), **(B)** (black: dominant phyla-Acidobacteria, Ascomycota, and Proteobacteria), and intermediate modules included **(C)** (blue: dominant with bacterial phyla Actinobacteria, Firmicutes, and Proteobacteria [*Streptomyces* spp., *Bacillus* spp. and *Pseudomonas* spp.] and fungi (*Ilyonectria* spp., *Fusarium oxysporum*, and *Mortierella* spp.), **(D)** (green: primarily *Galactomyces geotrichum*), **(E)** (yellow: primarily Firmicutes [*Paenibacillus* spp., and *C. saccharoperbutylacetonicum*]), **(G)** (red) and **(H)** (gray; fluctuating trends with unknown functional significance). Microbial proliferation module was comprised of **(F)** (brown: primarily Firmicutes [*Clostridium thiosulfatireducens*]). ASD was conducted using rice bran as the carbon source at a rate of 20 t ha^–1^. Weighted gene correlation network analysis (WGCNA) was used to generate signed network using soft threshold power = 10. Cytoscape Allegro Edge-Repulsive Fruchterman-Reingold layout was used to visualize the network. Nodes representing metabolites with highly correlated levels are the same color.

The metabolite network consists of 691 nodes and 125,539 edges with 6 modules (Figure 6). The microbial network is comprised of 5,634 nodes and 7,874,763 edges with 8 modules (Figure 7). The gray modules of both metabolite and microbial networks are comprised of nodes that did not cluster. Based on power analysis, the metabolic and microbial networks satisfied scale-free criterion. The same module color between the metabolite and microbial networks does not indicate a similar change over time.

### Correlations Among Metabolite and Microbial Modules

Changes within the community metabolome that influenced successional changes in the soil microbiome were clearly indicated by correlations over time among metabolite and microbial module eigenvalues (Table 1). The sequential transition through the three physiological phases (Phases 1, 2, and 3) during ASD-RB is evident given metabolic transitions related to consumption (Supplementary Figure S1) and production (Supplementary Figure S2).

**TABLE 1:** Correlation analysis of dynamics of change in Eigen vectors of microbial modules with metabolite modules during the 15-day incubation period of anaerobic soil disinfestation (ASD) signifying the manner of metabolite consumption and production by specific groups of microorganisms.

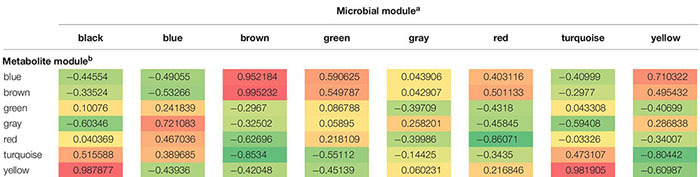

*Simulated anaerobic soil disinfestation treatment was carried out in microcosms using rice bran as the carbon source (equivalent to 20 Mg per ha per 15 cm depth in the field). Soil microbiome and metabolome networks were generated using weighted gene correlation network analysis (WGCNA) to capture the temporal dynamics of the system. Module Eigen vectors of each microbial and metabolite module signifying dynamics of relative levels of metabolites and abundance of microbial operational taxonomic units (OTUs) was used in the correlation analysis. ^*a*^Microbial modules were generated using WGCNA package with soft threshold power of 14, and dendrogram tree cut height of 0.45. ^*b*^Metabolite modules were generated using WGCNA package with soft threshold power of 10 and dendrogram tree cut height of 0.15. Color scheme represents signed value of the Pearson’s correlation between two arrays of module Eigen vectors of given two modules. Color designations of metabolite and microbial modules are independent and are not correlated.*

#### Phase 1

Aerobic microbial proliferation and decline in response to atmospheric modification were related to rapid O_2,_ amino acid (alanine, serine, threonine, and glutamic acid), and mono-, di-, and tri- saccharide (D-ribose, D-fructose, D-glucose, sucrose, and raffinose) consumption indicated by the yellow and turquoise metabolite modules (Figures 6A,B and Supplementary Figures S3, S4) and turquoise, black and blue microbial modules (Figures 7A–C and Table 1).

The microbial turquoise module (3839 OTUs) contains microbes that proliferate alongside the reduction of compounds residing in the yellow and turquoise metabolic modules. This microbial module consisted predominantly of aerobic bacterial and fungal phyla (Supplementary Table S2). The aerobic Firmicutes *Bacillus* and *Pasteuria* spp. along with members of Ascomycota belonging to the genera *Penicillium* and *Acremonium* were prominent in the turquoise module. The blue microbial module contains both aerobes and facultative anaerobes. Among the bacterial phyla present in blue module that consume amino acids, Actinobacteria, Firmicutes, and Proteobacteria were dominant with *Streptomyces* spp., *Bacillus* spp., and *Pseudomonas* spp. and *Pasteuria* spp. being significant representatives, respectively. Examples of important fungal plant pathogenic members of blue module are *Ilyonectria* spp. and *F. oxysporum.* Dominant phyla in the black module were Acidobacteria, Ascomycota, and Proteobacteria. Among the Ascomycetes, potential biocontrol agents such as *Chloridium* sp. (Kharwar et al., 2009) and *Trichoderma koningii* (Simon and Sivasithamparam, 1988) were exclusively present in black module.

### Phase 2

Atmospheric conditions favoring facultatively anaerobic microbes developed as oxygen was consumed by strict aerobes. Lactic acid and ethanol (gray and blue metabolite modules, respectively) levels peaked on day 2 (Supplementary Figures S4, S5) delineating this phase. Microbial populations linked with this phase increased until O_2_ levels reached a suboptimal level (blue microbial module). Facultative anaerobes that thrived during this period included Firmicutes, Actinobacteria, Proteobacteria and Zygomycota.

Levels of *Galactomyces geotrichum* (green microbial module; Figure 7D) was found in ASD-RB treated soils at greatest abundance during this phase of incubation. Lactic acid (gray metabolite module; Figure 6C) fermentation correlated with proliferation of *Bacillus* spp. Zygomycota was almost exclusively present in the blue module with *Mortierella* spp. being the only genus detected. *Mortierella* spp. increased from 2.04% at incubation duration zero to 35.1% at day 1, but then declined rapidly to 2.04% by day 3 (Supplementary Figure S6).

Many metabolites produced during phase 2 resided in the blue metabolite module and they continued to be produced during the subsequent phase. Consequently, these metabolites may be important during both phases 2 and 3. The metabolic profile included products of butyrate fermentation, acetone-butanol fermentation and homoacetic acid fermentation (acetone, acetic acid, 1-butanol, and butanoic acid) which began to accumulate alongside proliferation of various species of Clostridia known to carry out these reactions (Müller, 2001). Levels of metabolites in the blue metabolite module were temporally correlated with the microbial yellow module (Table 1) indicating that *Clostridium saccharobutylicum*, *C. saccharoperbutylacetonicum*, and *Paenibacillus* spp. (Figure 7E) began to proliferate and produce various small chain fatty acids and alcohols (e.g., 1-butanol, 2-butanol; Keis et al., 2001).

#### Phase 3

This phase is marked by the proliferation of strict anaerobes and their metabolic products (Supplementary Figures S5, S7), many with known antimicrobial properties. Firmicutes in microbial brown module were amplified at this anaerobic stage. The Firmicutes community continued to demonstrate dynamic changes in composition during this period and represented the dominant bacterial phylum detected in ASD-RB treated soils. Proliferation of Firmicutes has been documented repeatedly in response to ASD, however, prior to this study, changes in composition of this population over the course of the ASD treatment have not been detailed.

Three metabolic modules were associated with this phase (turquoise, blue, and brown modules; Figures 6B,D,E). Levels of many triacylglycerides and a monogalactosyldiacylglycerol in the metabolic turquoise module (Supplementary Figure S4) were inversely associated with microbial levels in the brown module (Figure 7F) indicating gradual consumption of lipids and accumulation of byproducts such as glycerol, organic acids, aldehydes, alcohols in the blue module and hydrocarbons residing in brown module (Supplementary Figures S5, S7, respectively). Non-polar metabolites indicative of phytosterols were also observed in the brown module.

**FIGURE 8:**
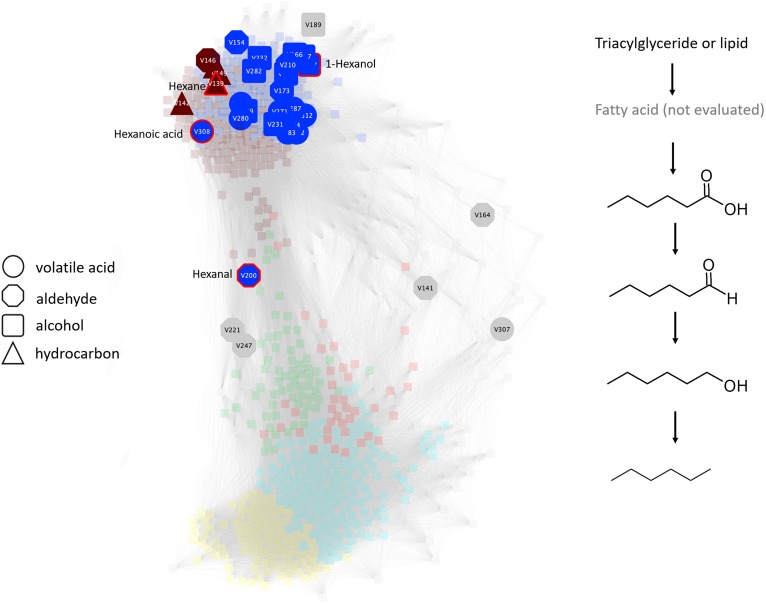
Undirected pairwise correlation network (left) highlighting volatile acids, aldehydes, alcohols, and hydrocarbons produced during simulated anaerobic soil disinfestation (ASD) established using rice bran amendment at a rate of 20 t ha^–1^. The WGCNA network determines network neighborhoods (brown and blue metabolic modules) associated with the latter, anaerobic stages of the process (also see Supplementary Table S1). Production of many of these metabolites is associated with this anaerobic stage and, where all metabolites are present as demonstrated by the production of C6 compounds (right), co-production may outline a putative pathway contributed by one or more community members of the transitioning microbiome. Triacylglycerides or glycerolipids, most of which are members of the turquoise module, are suggested as possible sources of substrate for volatile organic compound production. Compounds outlined in the putative pathway are highlighted in red in the network.

Microbes in the brown (*G. geotrichum, Penicillium* spp., *Pseudomonas* spp.), yellow (*G. geotrichum*), and green (*G. geotrichum, Pseudomonas* spp.) microbial modules that produce lipases are linked with the gradual consumption of lipids (Figure 7D). Additionally, *Clostridium thiosulfatireducens*, *C. peptidivorans*, and *C. tunisiense* in brown microbial module were highly correlated with the blue metabolite module which consisted of organic acids such as acetate and butyrate. *Clostridium scatologens*, *C. fimentarium C. populeti* were highly abundant by 15 days of incubation, the incubation duration point at which the organic acids acetate and butyrate were at peak accumulation. Production of these compounds is purportedly highest when atmospheric concentrations are maintained at O_2_ < 4% and CO_2_ > 19.2% (Runia et al., 2014). *Clostridium scatologenes* (brown microbial module) proliferation correlated with accumulation of *p*-cresol in blue metabolite module (Supplementary Figure 5E). Concerted changes in levels of metabolite groups in the blue and brown modules are indicative of lipid catabolism producing volatile fatty acids, aldehydes, alcohols (blue metabolite module members; Supplementary Figure S5), and volatile hydrocarbons such as pentane, n-hexane, heptane, and octane (brown metabolite members; Supplementary Figure S7) by a single or even multiple members of the anaerobic community (Figure 8). Production of dimethyl disulfide (DMDS), dimethyl trisulfide (DMTS), and dimethyl sulfoxide (Blue metabolite module; Supplementary Figure S5), were still increasing by the end of the test period. Simultaneously, *Clostridium* spp. abundance increased from 0.38% on day 0 to 13% on day 3, and 38% on day 15.

## Discussion

Mowlick et al. (2013b) established that a physiologically available carbon source was required to generate characteristic ASD effects on the soil microbial community and resulting soil-borne disease suppression and enhanced crop yield (Mazzola et al., 2018). Similarly, in the current study the absence of a carbon resource, but in the presence of water addition and exclusion of oxygen equivalent to that of the ASD-RB treatment, no significant changes to the microbiome or metabolome were observed. Likewise, in the absence of an anaerobic period in the post-rice bran amendment soil environment, no suppression of the Fusarium wilt pathogen, *F. oxysporum* f. sp. *fragariae*, and to the contrary its proliferation, was observed (Figure 1). Thus, the remainder of the discussion will focus on changes during the ASD-RB treatment.

### Microbial Generation of Anaerobic Environment

Soil atmospheric changes result in the preferential selection of different physiological groups of microorganisms (Ivanov, 2010). ASD-RB caused a decrease in oxygen over time as determined by soil redox potential resulting in development of an anaerobic environment due to accumulation of reduced chemical species (Mowlick et al., 2013b). Simultaneous precipitous decrease in headspace O_2_ concentration and increase in CO_2_ concentration was observed within the first 32 h of this study, which can be attributed to proliferation of aerobic microorganisms. Similar trends in O_2_ and CO_2_ concentrations were observed in an earlier field study following application of ASD using a commercially available plant-based amendment (Runia et al., 2014). Hence, reduced O_2_ concentration induced by elevated aerobic microbial respiration in response to the required carbon amendment drove the establishment of soil conditions typical of ASD. The substrate-induced microbial derived increase in CO_2_ production lead to rapid generation of the anaerobic environment. Rice bran, the ASD substrate used in this study, contains proteins, vitamins, minerals, lipase, and 12–23% fat (Singh and Sogi, 2016) as well as sugars and amino acids that are rapidly consumed by aerobic microorganisms.

### Successional Events in Soil Microbiome Composition

ASD-stimulated successional events in soil microbiome composition have received limited examination with a majority of studies employing single incubation duration-point analyses. Proliferation of Firmicutes has been documented repeatedly in response to ASD, a population which correspondingly has been inferred to possess a role in disease control outcomes (Mowlick et al., 2014); however, prior to this study, changes in composition of this population over the course of the ASD treatment have not been detailed. The dominant genera identified within the Firmicutes community in response to ASD has varied, most often reported as *Clostridium* spp. (Mowlick et al., 2013a) but also *Ruminococcus* and *Coprococcus* (Huang et al., 2015a), with the prevailing group appearing to be directed, in part, by carbon input type. Longer-term end point analyses of ASD treated soils have documented the elevated abundance of *Bacteroidetes* (van Agtmaal et al., 2015; Mazzola et al., 2018), a phylum that responds strongly to fluctuating redox potential. In the majority of studies, which have utilized such a single terminal sampling time point, *Clostridium* has been the major genus of Firmicutes detected in ASD treated soils at the end of the incubation period (Momma, 2008; Mowlick et al., 2013a; Huang et al., 2015a). It is imperative to examine transitional events in the ASD-derived soil microbiome at a finer scale in order to draw significance as to their roles in the process of ASD and their relation to the generation of metabolites functional in soil-borne disease suppression.

In the current study, the Firmicute population increased rapidly through 24 h and thereafter represented greater than 60% of the bacterial community in ASD treated soil for the remainder of the incubation period (physiologic phase 3). Runia et al. (2014) in a multi-incubation duration point sampling study documented amplification of Firmicutes soil density followed by a decline during ASD incubation while Liu et al. (2016) reported elevated Firmicute density throughout a 16-day incubation period. Findings from the current study were distinct from previous studies in the fact that proliferation of Firmicutes was instantaneous within 24 h followed by a steady level in relative abundance. More frequent sampling toward the beginning of the incubation, where rapid changes of the microbiome could be expected allowed capturing this change in dynamics of Firmicutes in the current study. This distinction between studies could derive from differential lability of the carbon source utilized. Compositionally, the Firmicutes population exhibited continuous transformation over the course of the incubation period, a feature which has not been documented previously. *Bacillus* and *Paenibacillus* spp. dominated the bacterial community at day 2, but these genera drastically declined by day 15. Conversely, the proportion of anaerobic Firmicutes, including *Clostridium* spp., in the bacterial community rapidly increased during the same incubation duration period. The transitional nature in microbiome composition can suggest operative functional modes of pathogen antagonism at specific incubation duration points during ASD.

### Correlations Between Altered Microbial Community Structure and Soil Metabolome

In the current study, detection of specific metabolites known to possess antimicrobial activity corresponded with amplification of specific microbial groups with capacity to yield such metabolites. Generation of VFA is considered a key chemical mode of action contributing to ASD efficacy (Momma et al., 2006; Runia et al., 2014) and *Clostridium* spp. are primary producers of these metabolites. Microbes in the brown (*G. geotrichum;* Burkert et al., 2005, *Penicillium spp.;* Pinheiro et al., 2008, *Pseudomonas* spp.; Kiran et al., 2008) and yellow (*G. geotrichum*), and green (*G. geotrichum, Pseudomonas spp.*) microbial modules that produce lipases are linked with the gradual consumption of lipids (Figure 6D). Bacterial catabolism of rice bran derived 16 and 18-carbon fatty acids under anaerobic conditions is possible, yet poorly described under anaerobic conditions (Roy et al., 1985; Kunau et al., 1995). Additionally, *Clostridium thiosulfatireducens*, *C. peptidivorans*, and *C. tunisiense* in brown microbial module can use proteins, peptides and amino acids to produce organic acids such as acetate and butyrate (Thabet et al., 2004), all of which are present in the highly correlated metabolite blue module. *Clostridium scatologens*, an acetogenic bacterium, can oxidize hydrogen to grow chemolithotrophically producing acetate from CO_2_ or CO (Song et al., 2014). Also, saccharolytic *Clostridium fimentarium* (Kotsiurbenko et al., 1995) can produce acetate, ethanol, and lactate, and cellulolytic *Clostridium populeti* (Sleat and Mah, 1985) can produce acetate, butyrate, and lactate.

Evidence for the yield of lipid catabolism products, such as VFAs, aldehydes, alcohols, and hydrocarbons, was documented by their presence in the blue and brown metabolic modules. Volatile hydrocarbons such as pentane, n-hexane, heptane, and octane, which were detected in the current study, have been linked to soil fungistasis (Lynch and Harper, 1974). Several yeast and bacterial species, including *Clostridium* spp., produce significant levels of hydrocarbons under anaerobic conditions (Ladygina et al., 2006). Various bacteria can reduce fatty acids to produce aldehydes and primary alcohols which are, in turn, dehydroxylated or decarbonylated to produce hydrocarbons (Ladygina et al., 2006).

Other compounds with demonstrable antimicrobial properties were also produced during phase 3. *p*-Cresol, is a microbiostatic compound produced in intestines, soils, and marine estuarine sediments (Heipieper et al., 1991). Only select bacterial species, including *Lactobacillus* sp., *Clostridium difficile*, and *C. scatologenes*, can produce *p*-cresol due to its toxicity (Scheline, 1968; Hafiz and Oakley, 1976; Yokoyama and Carlson, 1981). In this study, *Clostridium scatologenes* proliferation correlated with *p*-cresol accumulation. Production of dimethyl disulfide (DMDS), dimethyl trisulfide (DMTS), and dimethyl sulfoxide, which are antifungal and nematicidal volatile sulfide compounds (Hewavitharana et al., 2014), were still increasing in concentration by the end of the incubation period. *Clostridium* spp. were previously reported to produce DMDS (Stotzky et al., 1976). This finding has significance to the field application of ASD as it indicates the need for maintaining soil tarping, and corresponding retention of the anaerobic environment, for a period of 15 days or longer to achieve optimal levels of disease control.

Soil metabolism is a sequential process where one group of microorganisms transform carbon substrate, altering the environment for subsequent organisms. Since proliferation of one physiological group of microorganisms may be dependent on multiplication of another group(s) of microorganisms in a system like ASD, consideration of the community metabolome rather than metabolome analysis of a single culturable microorganism is important. This was clearly observed with respect to trends of metabolic modules. Some metabolic modules such as blue and brown modules showed increased production over time which paralleled the trend in microbial brown module indicating contribution of members of this microbial group (e.g., *Clostridium* spp.) in producing metabolites in the blue (ethanol, organic acids, and sulfides) and brown (hydrocarbons) modules. Another potential is that the decline of a particular metabolic group may indicate its consumption by a microbial module that proliferated inversely to the metabolic module. For instance, microbial brown module showed a gradual relative increase at the same time that metabolic turquoise module showed a gradual decrease which indicated consumption of primary metabolites such as amino acids and pyruvic acid. With this knowledge in hand, metabolic markers of single organisms could be further explored as it narrows down the vast number of possibilities to one or two modules.

### Implications for Use of ASD in Soil-Borne Disease Management

Disease management strategies that employ multiple mechanisms are key to sustaining a prolonged period of soil-borne pathogen control. The capacity of pathogens to circumvent the activity of a single mode of action is well known in terms of fungicide resistance (Deising et al., 2008) or loss of host resistance when conferred by a single dominant gene (Agrios, 2005). Practically, ASD can provide more durable disease control without soil fumigation (Mazzola and Manici, 2012). Our evidence indicates, on a systemic basis, that disease control may result from a combination of competition for substrate, selective substrate availability at critical incubation duration points during ASD progression and resulting production of multiple antagonistic metabolites. Field application of ASD has commonly employed a 2-3-week ASD incubation period (Momma et al., 2010; Mazzola et al., 2018). The temporal profiles of various biologically active metabolites detected suggest that modification of the application method to extend the incubation period may yield enhanced disease control efficacy. For instance, many biologically active metabolites, including acetic acid, valeric acid (pentanoic acid), dimethyl disulfide, dimethyl trisulfide and *p*-cresol, were detected at highest levels at the final sampling incubation duration point (15 days) and exhibited a trajectory that projected a continued increase. Prolonged incubation could ensure pathogen exposure to the period of maximum anti-microbial metabolite generation.

The majority of ASD related mechanistic studies has focused on presence or production of antibiotic or antagonistic metabolites that may also heavily depend on carbon source and environment. Just as food fermentations produce ethanol or lactic acid which, in turn, renders an environment toxic to foodborne pathogens, soil undergoing ASD produces a number of compounds toxic to plant pathogens resulting from a number of modes of action. The majority of these compounds produced during our ASD treatment resulted from metabolism during the anaerobic phase, possibly by microbes consuming lipids. As mentioned above, these compounds include short-chain fatty acids and alcohols, hydrocarbons, *p*-cresol, and alkyl sulfides, all having potentially different modes of action.

**FIGURE 9:**
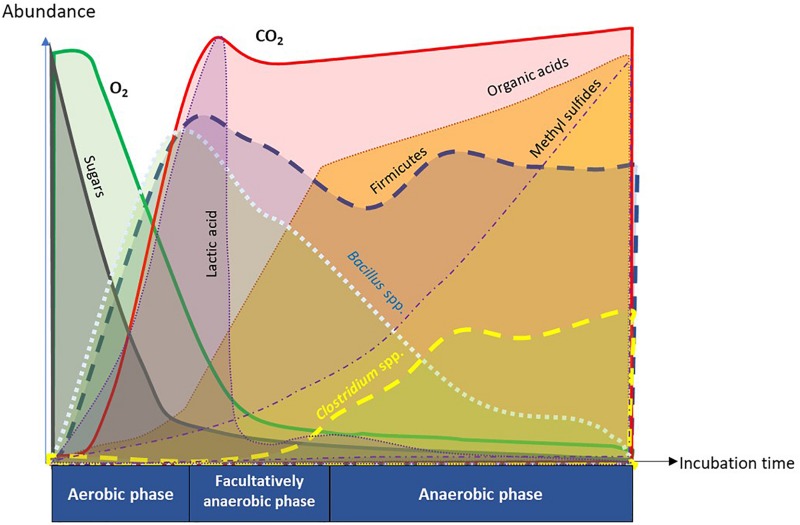
Summary diagram of key functional attributes of anaerobic soil disinfestation (ASD) discerned by temporal dynamics of soil metabolome and microbiome. ASD incubation consists of sequential changes in soil physiology from aerobic, facultatively anaerobic, to anaerobic phases in response to dynamics of O_2_ and CO_2_. Depletion of O_2_ in the soil atmosphere was caused by proliferation of aerobic microorganisms consuming easily degradable organic matter such as sugars. The trend of initial proliferation of *Bacillus* spp. followed by decline was inverse for *Clostridium* spp. in phylum Firmicutes. Population dynamics of *Clostridium* spp. was associated with temporal trends in generation of organic acids and methyl sulfides that are crucial in suppression of soil-borne pathogens.

The transition of soil metabolome and microbiome observed during ASD has similarities to that occurring in fermentation processes where the coordinated provision of media to establish conditions that govern the controlled, sequential progression of microbial population is critical (Figure 9; Bisson, 1999; Guzzon et al., 2015). Thus, composition of the microbial population, the rate of transitional chemical change in the organic substrate, and quality of the resulting product is a function of available nutrients and timing of availability (Guzzon et al., 2015). In the simulated ASD, simple sugars provided a readily available carbon source for aerobic microbes to rapidly establish a hypoxic atmosphere. A wide variety of carbon sources have been studied for ASD ranging from highly labile (molasses; Rosskopf et al., 2015), moderately labile (e.g., rice bran; Shennan et al., 2014, wheat bran; Momma et al., 2010, freshly mown grass; Hewavitharana and Mazzola, 2016), to recalcitrant (e.g., maize straw; Huang et al., 2015b). A less available carbon source or a dearth of essential nutrients at critical points in the process, such as amino acids, may slow or alter the course of this process and alter the ASD succession and outcome of disease suppression.

Production of antagonistic compounds may be coordinated by multiple species. “Community metabolism” (Jones et al., 2014) can indicate instances of microbial syntrophy where one partner provides a required compound for metabolism by another. Most anaerobic processes are considered to be syntrophic (Morris et al., 2013). ASD is a prime example of exploiting classical syntrophy in anaerobic degradation of complex compounds. Our results, in part, indicate one possibility in the current system where decreased lipid levels coincide with increased volatile acids, aldehydes, alcohols, and hydrocarbons and proliferation of multiple microbial species that could have contributed to one or more reactions. More importantly to disease control, it emphasizes the potential importance of the presence and specific metabolism of any single species involved in the production of multiple classes of antimicrobial compounds.

## Conclusion

Carbon source is a key factor that drives changes in soil chemistry, microbiome composition and metabolic transformations during the ASD-RB treatment. Time-series analysis of the microbiome and metabolome allowed for detection of both gradual and punctuated changes in the soil system while highlighting associations between microbial and metabolite modules that potentially contribute to soil-borne disease suppression. While previous studies have focused on both biological and chemical modes of disease suppression, limitations such as use of a single terminal sampling incubation duration point, sole focus on organic acids, or changes only in bacterial populations hindered overall understanding of functional attributes of ASD. In the current study, these aspects were addressed, and potential new modes of action (i.e., fungistatic features of hydrocarbons and *p*-cresol) were suggested. Findings in this study provide more direction for field application of ASD. For instance, the significance of labile carbon source to increase the rate of attainment of anaerobicity and minimum incubation period duration for generation of certain chemical modes of actions, such as methyl sulfides, have been elucidated.

## Data Availability Statement

The raw data supporting the conclusions of this manuscript will be made available by the authors, without undue reservation, to any qualified researcher.

## Author Contributions

SH designed and performed the experiments, conducted the data analyses, and wrote the manuscript. EK performed *Fusarium oxysporum* f. sp. *fragariae* disease assessment experiments. AR contributed to designing the experiments and conducting preliminary experiments. RL conducted the volatile analysis experiments. BP contributed to annotating non-polar metabolites. LH contributed to access high-end computing for network generation and visualization, and programing to generate microbial and metabolite networks. DR contributed to insightful discussions on designing the project, metabolite analyses, data analyses, network generation, and manuscript writing. MM supervised the entire project including microbiome analyses experiments, data analyses, interpretation of the findings, and writing of the manuscript. All authors read and revised the manuscript.

## Conflict of Interest

The authors declare that the research was conducted in the absence of any commercial or financial relationships that could be construed as a potential conflict of interest.

## Footnotes

1 http://rdp.cme.msu.edu/

2 www.ncbi.nlm.nih.gov
