# Supplementary material for: Temporal Dynamics of the Soil Metabolome and Microbiome During Simulated Anaerobic Soil Disinfestation
Source: Front Microbiol. 2019 Oct 15;10:2365. doi: 10.3389/fmicb.2019.02365 (PMC6803440; doi:10.3389/fmicb.2019.02365)
Supplement: Supplementary file 3 [file Data_Sheet_3.docx]

Supplementary Material

# Supplementary Figures and Tables


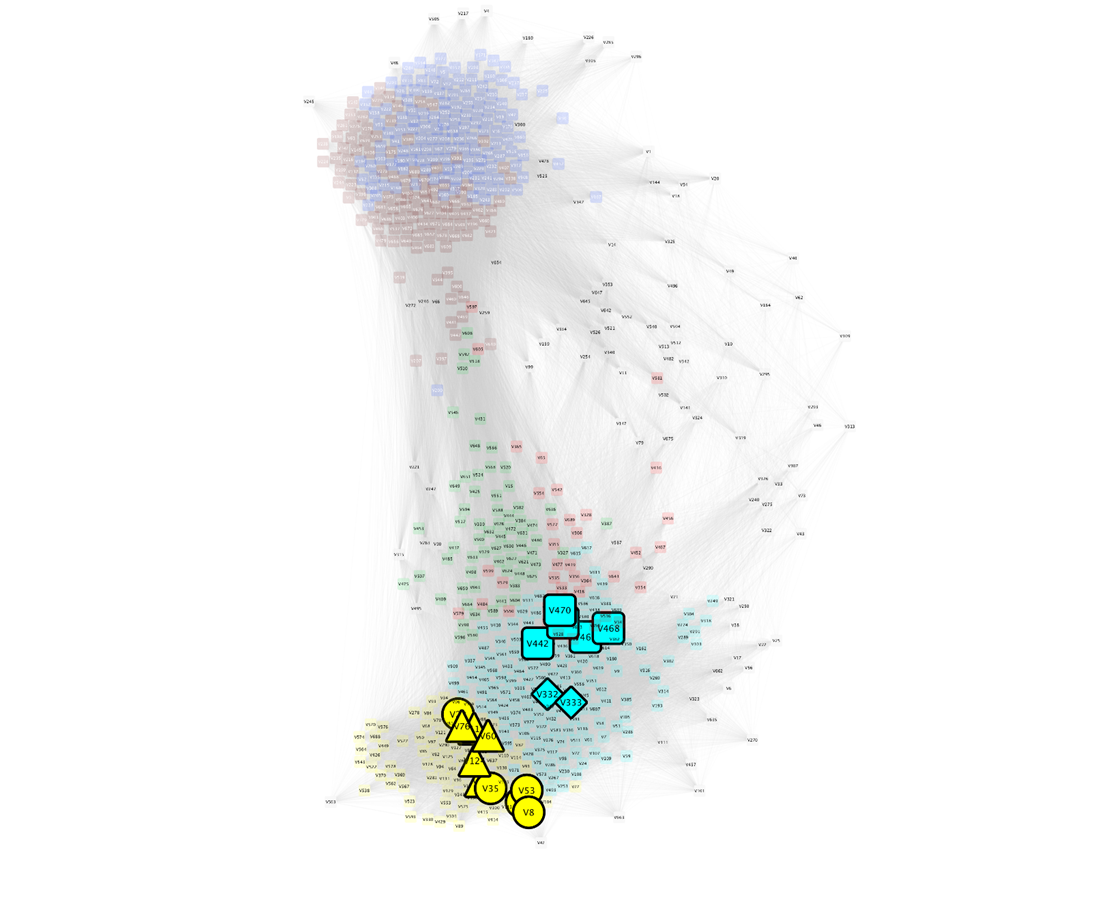


**Supplementary Figure 1.** Consumption metabolite network in anaerobic soil disinfestation (ASD-RB) treated soil with rice bran. Yellow and turquoise metabolite module members were immediately consumed by aerobic microorganisms. Lability of the carbon source consumed by the microorganisms may have impact on the required duration of the ASD treatment. The more labile the carbon source is, the faster the occurrence of sequential phases of ASD-RB. Composition of the carbon source such as amino acids, sugars, and lipids governs the lability and ultimately factor in production of disease suppressive metabolites in soil. Highlighted yellow module members are amino acids (circles), sugars (triangles) and turquoise module members are triacyl glycerides (squares) and monogalactosyldiacylglycerol (diamonds).

**
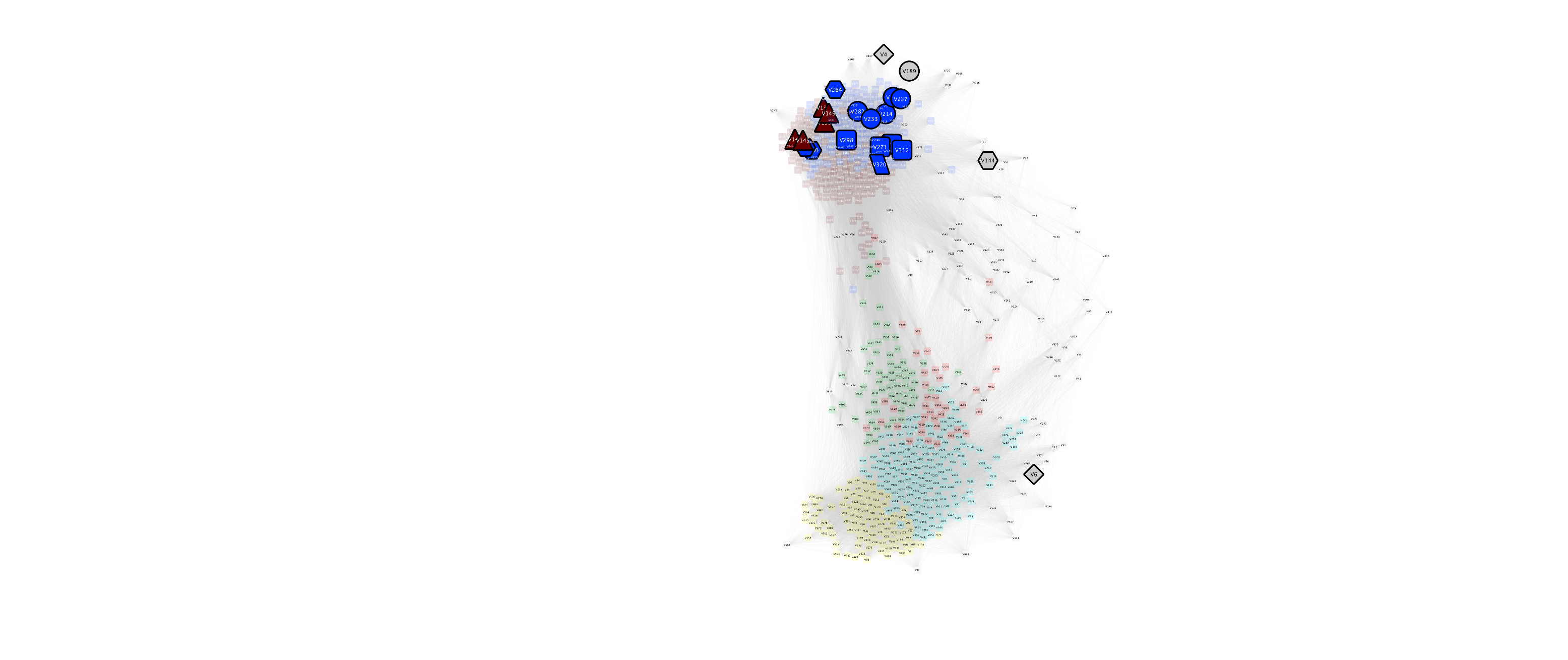
Supplementary Figure 2.** Production metabolite network in anaerobic soil disinfestation (ASD-RB) treated soil with rice bran. Metabolites that accumulated over time were produced by various microorganisms. Several metabolites such as lactic acid and pyruvic acid provide evidence for occurrence of microbial fermentation. Other metabolites such as *p*-cresol, volatile organic acids, hydrocarbons, and methyl sulfides are the determinants of disease suppressiveness by ASD. Highlighted blue module members are *p*-cresol (rhombus), volatile organic acids (squares), alcohols (circles), methyl sulfur compounds (hexagons), brown module members are hydrocarbons (triangles), and gray module members are primary metabolites (squares), and dimethyl sulfide (hexagon).


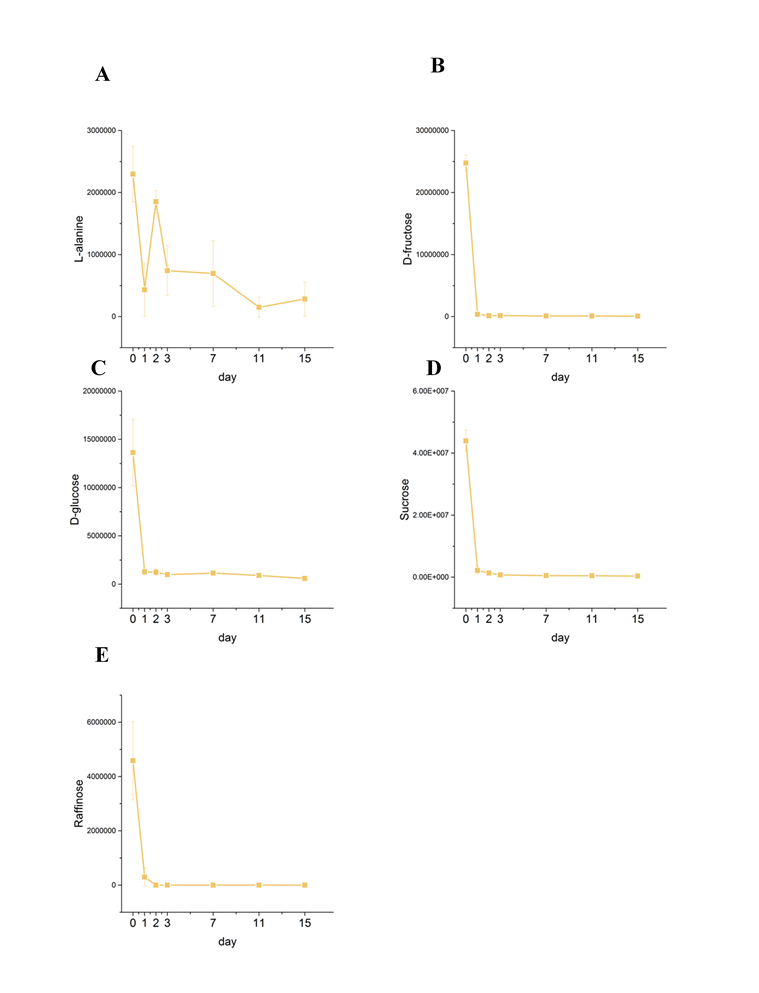


**Supplementary Figure 3.** Change in relative level of selected individual metabolites of yellow (consumption) module which included amino acids and sugars. Trends of the individual metabolites approximately followed the trend of the Eigen vector of yellow module. **A**: L-alanine, **B**: D-fructose, **C**: D-glucose, **E**: Sucrose, and **F**: Raffinose. Error bars represent standard error of the mean with n = 4.


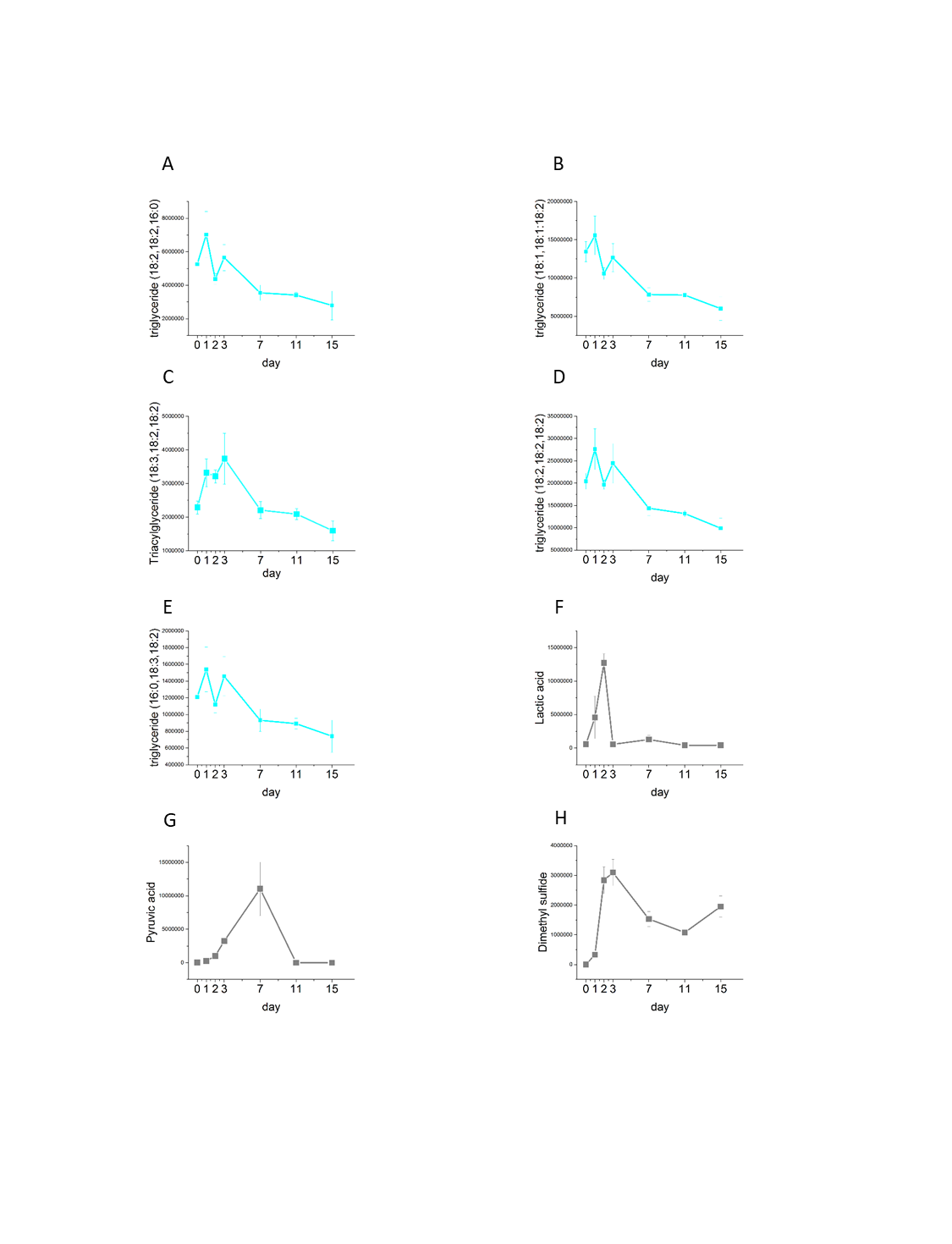


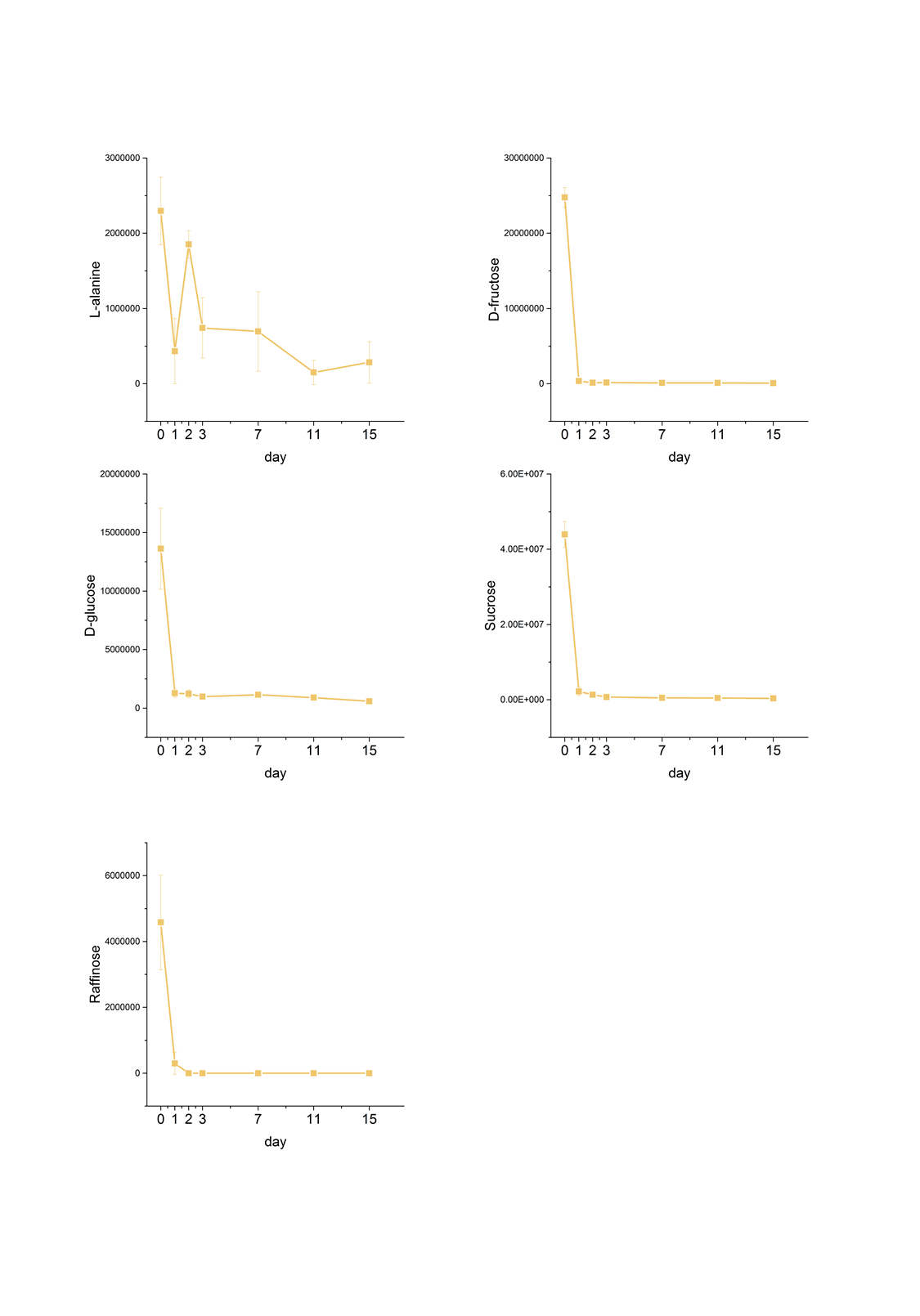


**A**

**B**

**C**

**D**

**E**

**Supplementary Figure 4.** Change in relative level of select individual metabolites of turquoise module that contains triacylglycerides and gray module that contains unassociated metabolites including primary metabolites and a methyl sulfide. Dynamics in abundance of the individual metabolites approximately followed the trend of the Eigen vector of turquoise and gray modules. **A**: triglyceride (18:2, 18:2, 16:0), **B**: triglyceride (18:1, 18:1, 18:2), **C**: triglyceride (18:3, 18:2, 18:2), **D**: triglyceride (18:2, 18:2, 18:2), **E**: triglyceride (16:0, 18:3, 18:2), **F**: Lactic acid, **G**: Pyruvic acid, and **H**: Dimethyl sulfide. Error bars represent standard error of the mean with n =4.


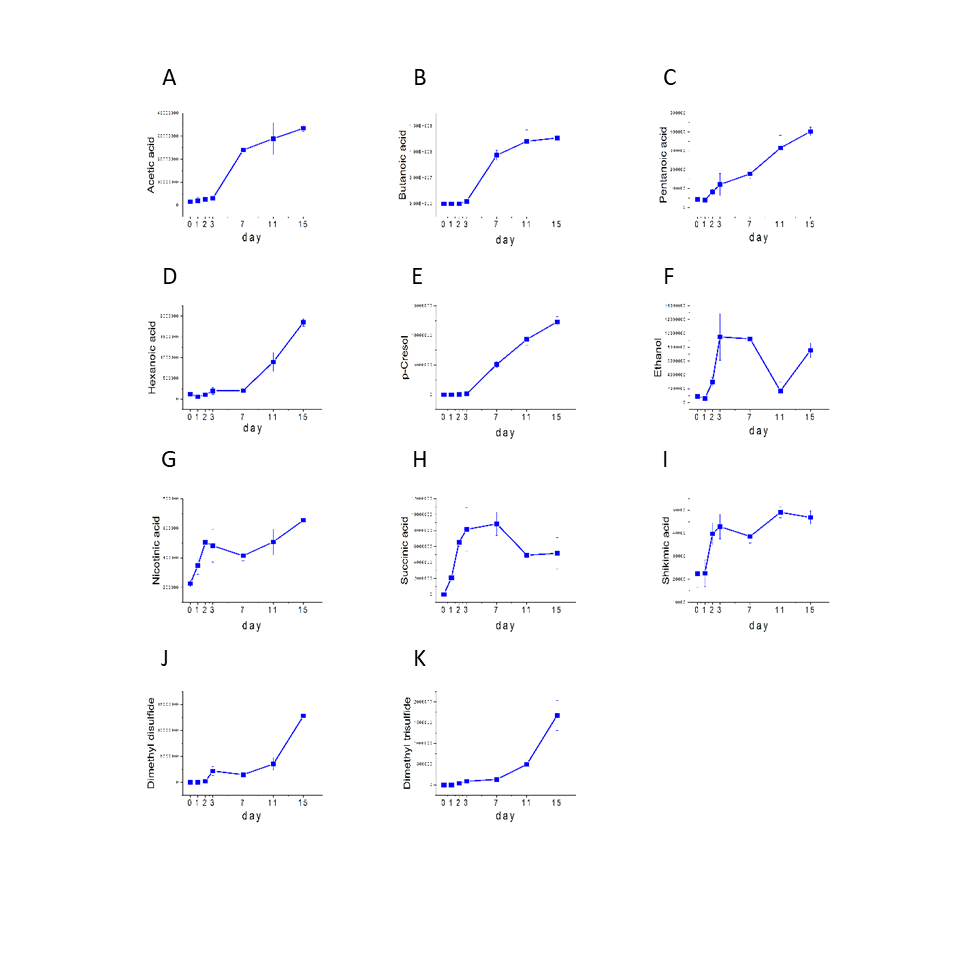


**Supplementary Figure 5**. Change in relative level of select individual metabolites of blue module. Blue module included organic acids, *p*-Cresol, ethanol, and primary metabolites. Dynamics of abundance of the individual metabolites approximately followed the trend of the Eigen vector of blue module. **A**: Acetic acid, **B**: Butanoic acid, **C**: Pentanoic acid, **D**: Hexanoic acid, **E**: *p*-Cresol, **F**: Ethanol, **G**: Nicotinic acid, and **H**: Succinic acid, **I**: Shikimic acid, **J**: Dimethyl disulfide, and **K**: Dimethyl trisulfide. Error bars represent standard error of the mean with n = 4.

**Supplementary Figure 6.** Changes in relative abundance of fungal taxonomic divisions over a period of 15 days in the anaerobic soil disinfestation treatment with rice bran. ASD = anaerobic soil disinfestation using rice bran as the carbon source at a rate equivalent to 20 t ha^-1^ for 15 cm depth.


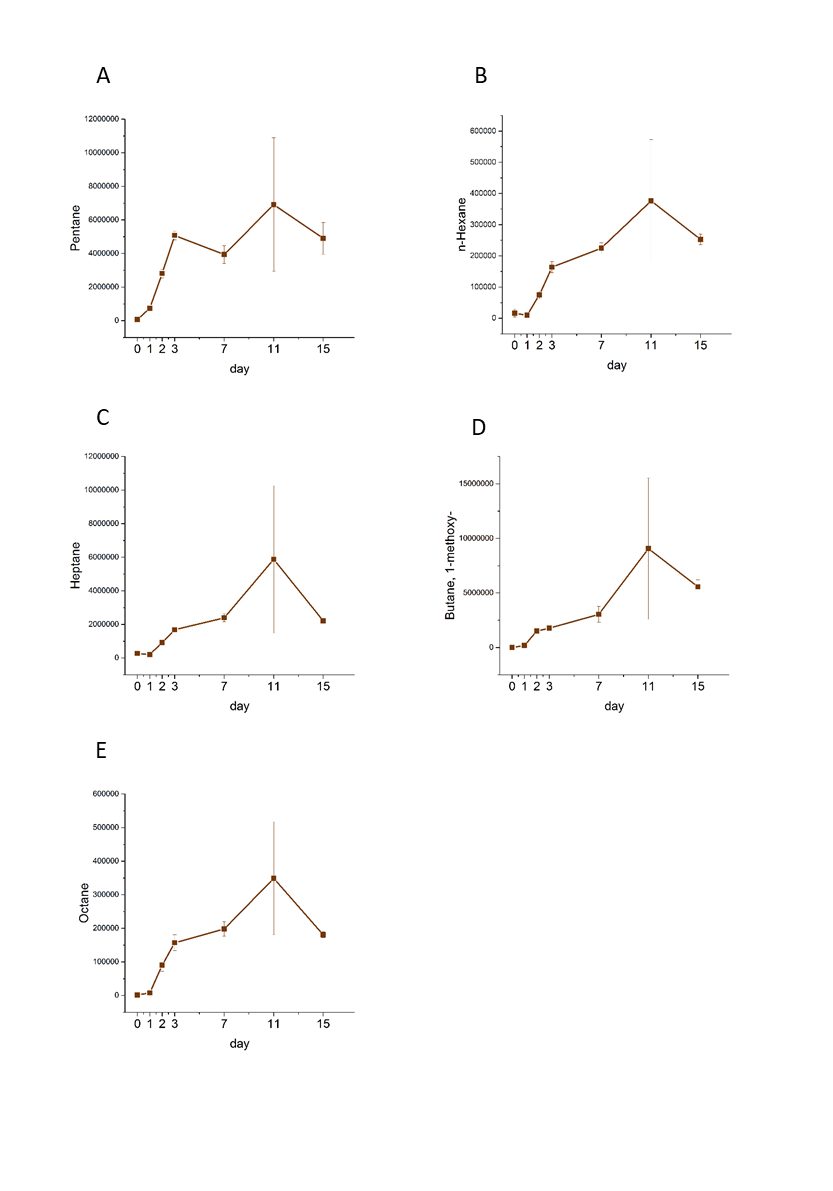


**Supplementary Figure 7.** Change in relative level of select individual metabolites of brown module which included hydrocarbons. Dynamics of abundance of the individual metabolites approximately followed the trend of the Eigen vector of brown module. **A**: Pentane, **B**: n-Hexane, and **C**: Heptane, **D**: 1-methyoxy, and E: Octane. Error bars represent standard error of the mean with n = 4.
